# Improving Physical Health Monitoring on a Working-Age General Adult Inpatient Ward: A Quality Improvement Project

**DOI:** 10.1192/bjo.2025.10408

**Published:** 2025-06-20

**Authors:** Mohsin Nazeer Muhammed, Abeer Jawed, Nadarasar Yoganathan

**Affiliations:** 1 Surrey and Borders Partnership NHS Foundation Trust, Surrey, United Kingdom; 2 Central and North West London NHS Foundation Trust, London, United Kingdom

## Abstract

**Aims:** Individuals with severe mental illness (SMI) face a significantly reduced life expectancy, primarily due to preventable physical health conditions. This project aimed to enhance the timeliness and comprehensiveness of physical health checks for inpatients in an acute psychiatric ward. An initial audit cycle identified gaps, prompting targeted interventions, with subsequent re-audit assessing their impact.

**Methods:** The first audit cycle (March 2023) reviewed adherence to physical health assessments, including physical examinations, observations, height and weight measurements, ECGs, blood tests, and cardiometabolic checklist completion. Interventions implemented included daily reviews in the Multi-Disciplinary Team (MDT), integration into daily job lists, and master list documentation. These measures reduced delays in completing assessments.

The second cycle (November 2023) involved 35 inpatients over four months. Interventions included the introduction of a whiteboard for task tracking, post-MDT reviews, staff reminders, and induction sessions emphasising physical health monitoring. Colour coding was introduced to enhance task visibility and efficiency. Specific patient needs, such as those with heart failure and left bundle branch block (LBBB) or end-stage renal disease (ESRD) requiring dialysis, were incorporated into tailored care plans.

**Results:** The second cycle demonstrated that, overall, there were visible improvements in clinical practice. The whiteboard intervention significantly improved the timeliness and completion rates of physical health checks. Key findings included:

Physical examinations: Success rates increased from 93% to 100%.

BMI measurements: Reduced delays and increased completions.

Physical observations: Maintained at 100% completion.

Challenges included gender-based refusals for ECGs and reluctance from patients with eating disorders to undergo BMI measurements. These findings highlight the importance of personalised approaches to monitoring and addressing barriers to compliance.

**Conclusion:** Implementing a whiteboard for tracking physical health checks demonstrated substantial improvements in timeliness and completion rates through simple, cost-effective interventions. Despite challenges, this project underscores the potential of structured systems to enhance physical healthcare for patients with SMI. Scaling and expanding these strategies hospital-wide may contribute to addressing health disparities and improving outcomes for this vulnerable population.

